# Proprioception After Multiligament Knee Injury: Does Ligament Repair Lead to Better Proprioceptive Acuity Than Ligament Reconstruction?

**DOI:** 10.7759/cureus.11380

**Published:** 2020-11-08

**Authors:** Hannah L Burton, Jon R A. Phillips, Nitin P Badhe, Benjamin J Olliverre, Christopher G Moran

**Affiliations:** 1 Trauma and Orthopaedics, Royal Devon and Exeter NHS Foundation Trust, Exeter, GBR; 2 Trauma and Orthopaedics, Queen's Medical Centre, Nottingham, GBR

**Keywords:** proprioception, multiligament knee injury, knee dislocation, ligament reconstruction, ligament repair

## Abstract

Introduction

Multiligament knee injuries are uncommon but serious injuries. There is ongoing debate on the optimal treatment of these injuries. We designed a study to establish the effects of repair or reconstruction on proprioceptive outcomes following multiligament injury to the knee.

Materials and Methods

A total of 34 patients were analysed by independent researchers who had no conflict of interest in the cases (23 in the repair group and 11 in the reconstruction group). Proprioception of the knee was measured using a previously validated tool to assess the reproduction of passive positioning. Functional outcome was measured using the Lysholm score. Sub-group analysis was performed. The mean time from injury to review was 83 months (range: 25-193 months).

Results

There were no significant differences in proprioceptive acuity between the injured (5.9±4.2°; range: 1.0-18.3°) and uninjured contralateral (control) knees (5.2±3.8°; range: 1.0-15.0°; p=0.35). Similarly, there was no significant difference in proprioceptive acuity identified between the injured knees that underwent repair (6.0±4.3°; range: 1.0-18.3°) or reconstruction (5.0±3.6°; range: 1.3-14°; p=0.53).

Overall knee outcomes were good; the mean Lysholm score at final follow-up was 75.5±16.8 (range: 36-100). No significant differences were identified in any of the sub-groups.

Conclusions

We were unable to identify any differences in knee proprioceptive acuity between injured knees and controls nor between the types of surgical treatment, demonstrating equivocal recovery for both methods of treatment.

## Introduction

Multiligament knee injuries are uncommon but serious injuries that have historically been associated with poor clinical outcomes [1]. There clinical incidence is less than 10 in 10,000 of trauma cases [2]. By definition, they are an injury that involves damage to two or more ligaments of the knee that control knee stability; these include the anterior cruciate ligament (ACL), posterior cruciate ligament (PCL), medial collateral ligament, lateral collateral ligament, posteromedial corner and posterolateral corner. There is significant debate on the optimal treatment of these injuries, and decision-making is complicated by the variety of anatomical injury combinations, association with polytrauma and the frequency of concomitant vascular or nerve injuries [3-7].

Non-operative treatments have been associated with poor outcomes [1,8]. The principles of treatment currently revolve around either repairing the injured structures, reconstructing the injured structures using grafts or a combination of both. Two studies have demonstrated higher rates of treatment failure after isolated repair when compared to ligament reconstruction [6,9].

Proprioception is the conscious and unconscious perception of joint movement and joint position [10]: the sum of kinaesthesia (the sensation of limb position and movement) and joint position sense. There are proprioceptors present throughout the knee, such as mechanoreceptors present in the skin, muscles, tendons, ligaments and joint capsule, and kinaesthetic receptors present in the muscle spindles in the muscles around the knee. Abnormal knee proprioception has been identified in cases of both ACL and PCL deficiency, and abnormal knee proprioception is present in osteoarthritis. Proprioceptive acuity is improved after both ACL and PCL reconstruction, and proprioceptive training is now one of the key concepts in the rehabilitation of knees after ACL reconstruction [11].

The global effects of injury and surgical approach on the outcomes after multiligament knee injury are not known and neither are the effects of treatment. The two competing surgical doctrines of repair or reconstruction are likely to have different effects on proprioception, and proprioception itself is likely to determine to an extent functional outcome. We designed a study to establish the effects of repair or reconstruction on proprioceptive outcomes following multiligament injury to the knee.

## Materials and methods

Following regional ethical approval, patients were retrospectively identified from a prospective trauma database at the Queen’s Medical Centre, Nottingham, UK. Patients were included in the study if they had sustained two or more ligament injuries or a posterolateral or posteromedial corner injury following trauma to the knee. Prospective follow-up was undertaken in a research clinic setting by an independent researcher. Injuries were proven by MRI scan, and all patients were managed initially according to the ATLS (Advanced Trauma Life Support) guidelines and local limb salvage policy. Vascular injuries were managed in conjunction with the vascular surgeons and initial reduction, and first aid took place in the resuscitation room. Patients were included in the study if definitive fixation occurred within four weeks of diagnosis, and patients were only included in the study if they were able to attend a research clinic.

All patients underwent a standardised assessment including review by one of two independent researchers who had not previously been involved in patient care. Hospital notes were retrieved, and information was collected on the diagnosis, pattern of injury and type of treatment performed.

All patients had undergone treatment of their multiligament knee injury at the Queen’s Medical Centre. Two surgeons performed all of the surgeries. One surgeon (C. G. M.) performed acute anatomical surgical repair of injured structures, whereas the other surgeon (N. P. B.) used a method published by Engebretsen [12], performing ligament reconstruction with repair of certain structures.

Surgical anatomical repair of all injured structures using bone anchors and sutures was undertaken in a stepwise method, which included meniscal repair and direct repair or reattachment of the posterior capsule to the midline. The PCL was repaired when injured, but ACL reconstruction was undertaken using a bone patellar bone autograft at the same sitting or at six months post-repair, depending on injury pattern.

Ligament reconstruction of medial, posteromedial and posterolateral structures, with associated soft tissue repair was undertaken in a stepwise manner with synchronous PCL and ACL reconstruction (depending upon the injury pattern, a delayed ACL reconstruction was performed). Ligament reconstruction was achieved with contralateral hamstring autograft (PCL), artificial graft (LARS ligament, Corin, England; posterolateral or medial) and ipsilateral bone patellar bone graft (ACL).

Patients underwent a structured interview where further information was collected about the injury, including complications or further surgery. The Lysholm knee outcome score was administered [13], and knee proprioception was then assessed using a modified Tornvall Chair, measuring the ability to reproduce passive positioning (RPP).

Classification of the injury according to Schenck was performed [14] using operative notes and findings on examination under anaesthetic and MRI outcomes.

The primary outcome measure was the measurement of proprioceptive acuity of the injured knee compared to the uninjured (control) knee, with secondary outcomes measure the Lysholm knee score. Predefined sub-group analysis included stratification by type of surgery (repair or reconstruction), sex, age, involvement in polytrauma, those who underwent further surgery and Schenck classification of injury [14].

Patients were assessed using a previously validated method using a Tornvall chair [15]. Patients were positioned on the apparatus, and passive positioning of the leg was performed at multiple pre-defined knee angles (the criterion angle) using a protractor. Visual and cutaneous stimuli were eliminated, and a counterbalance was used. The patient was then asked to reproduce this angle, and the reproduced angle was measured. The mean difference between the criterion and reproduced angle was taken as a measure of proprioceptive acuity.

Statistical analysis was performed using GraphPad prism Version 6.00 for Windows (GraphPad Software, La Jolla, CA, USA). Paired or unpaired Student’s t-tests were used to analyse continuous data, and statistical significance was taken at 95% in all cases. Post-hoc power analysis was performed using G*Power 3.1.9.2 (University of Dusseldorf, Dusseldorf, Germany) in order to stablish a power of >0/8 with a significance level of <0.05 [16].

## Results

**Figure 1:**
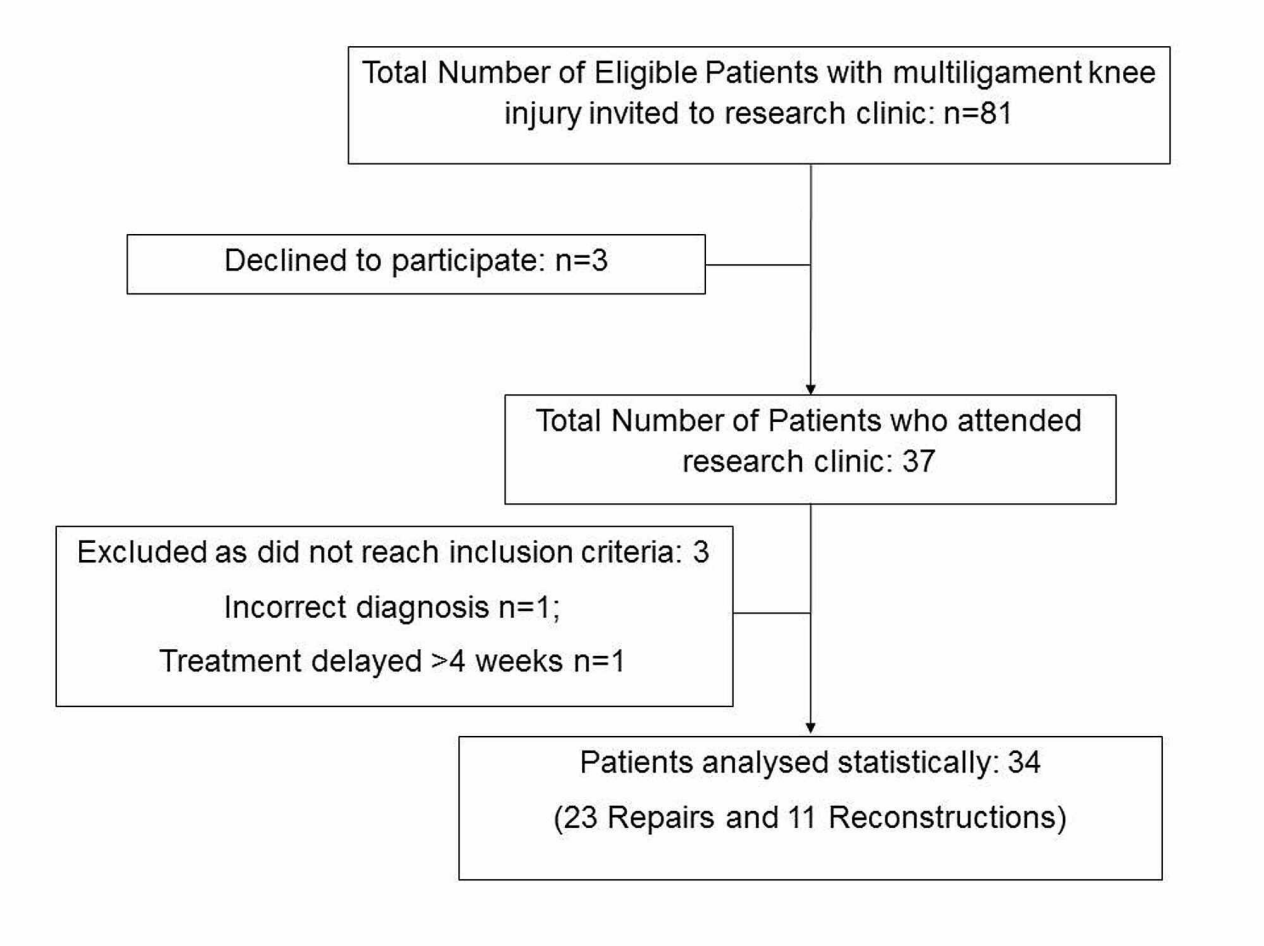
Flowchart of subject recruitment for study

In total, 34 patients completed the study and formed the basis of this report. A total of 81 patients were identified from the prospective trauma database who were eligible for participation. Figure 1 demonstrates the flowchart for patient recruitment. Patient demographics are demonstrated in Table 1. The mean time from injury to follow up was 83±47 (standard deviation) months (range: 25-193 months). Of the 34 patients, 23 (68%) underwent surgical repair and 11 (32%) underwent ligament reconstruction (with repair); there was a broad representation of Schenck types (Table 2).

**Table 1:** Demographics of patients in each surgical treatment group *Not statistically significant (p=0.06)

	Total	Repair	Reconstruction
Total number	34	23	11
Age at time of injury (years)			
Mean	37.2	37.2	37.2
SD	12.6	11.6	14.8
Range	17-62		
Side	19 (56%) right	15 (65%) right	4 (36%) right
Sex	11 (32%) female	6 (26%) female	5 (45%) female
Days from injury to surgery			
Mean	7	6	8
SD	5	4	6
Range	1-23	1-19	1-23
Months of follow-up			
Mean	83	94	61*
SD	47	50	37
Range	25-193	25-193	26-153

**Table 2:** Schenck classification of injuries, including a breakdown of the method of surgical treatment

Schenck	Total	Repair	Reconstruction
I	16	10	6
II	0	0	0
III Lateral	7	5	2
III Medial	3	2	1
IV	6	4	2
V	2	1	1

There were no significant differences in proprioceptive acuity between the injured (5.9±4.2°; range: 1.0-18.3°) and uninjured contralateral (control) knees (5.2±3.8°; range: 1.0-15.0°; p=0.35). Similarly, there was no significant difference in proprioceptive acuity identified between the injured knees that underwent repair (6.0±4.3°; range: 1.0-18.3°) and reconstruction (5.0±3.6°; range: 1.3-14°; p=0.53) (Table 3).

**Table 3:** Proprioceptive acuity after multiligament knee injury, including comparison between the repair and reconstruction group

Proprioceptive acuity (degrees)	All	Repair	Reconstruction
N	34	23	11
Injured			
Mean	5.9	6.0	5.0
Median	4.8	4.7	4.7
SD	4.2	4.3	3.6
Range	1-18.3	1-18.3	1.3-14
Uninjured			
Mean	5.2	5.1	5.1
Median	4	4	4
SD	3.8	3.4	4.1
Range	1-15	1-17.3	1-15

The predetermined sub-groups were analysed, and no significant differences in proprioceptive acuity for the injured knees between men and women were identified, and nor was a difference found between the injured and uninjured knees in either the male or female groups. Proprioceptive acuity was, however, significantly better in the uninjured knee in men than women (4.2±2.4° vs 6.9±5.0°; p=0.048).

Again, there was no significant difference found in the injured knees in either of the age groups or between age groups. Proprioceptive acuity of the uninjured knees in younger patients (<26 years) was better than that of the older patients (>40 years) (p=0.025). There were no differences when analysis was performed by injury mechanism or pattern. No difference in outcomes were found in patients who had been involved in polytrauma, predominantly medial versus lateral injuries, in the Schenck classification, or whether the patient had undergone further surgery after their initial treatment.

Overall knee outcomes were good (Table 4); the mean Lysholm score at final follow-up was 75.5±16.8 (range: 36-100). No significant differences were identified in any of the sub-groups.

**Table 4:** Outcome score results at final follow-up, with comparison between the repair and reconstruction groups and statistical analysis

	Total	Repair	Recon
Total	34	23	11
Lysholm			
Mean	75.5	74.1	78.9
SD	16.8	18.9	10.1
Range	36-100	36-100	65-95

## Discussion

We were unable to identify any differences in knee proprioceptive acuity between injured knees and controls or between the types of surgical treatment.

To our knowledge, there are no published studies that have examined the proprioceptive acuity of knees after multiligament knee injury. There are many structures in the knee that contribute to knee proprioception; therefore, it was surprising that no difference was found between the injured and control knees. This leads to the conclusions that there is no difference between the groups and the null hypothesis is correct; alternatively, either the null hypothesis is incorrect or the tool for measuring knee proprioceptive acuity is not sensitive enough to detect a difference between the groups.

Due to the retrospective nature of this study, no power analysis was included in its design. A post-hoc power analysis was performed, and with the results from the study, a total sample size of 236 patients would be required to ensure there is no B error. Post-hoc power calculations have been widely used in the literature, but contemporary thinking suggests that these are potentially flawed [17] and that as such any post-hoc power analysis must be viewed with caution. We acknowledge the small sample size of this study and the potential for influence of chance, but our results have shown there is a basis for future research in this field. It is also very difficult to assess each injury combination in isolation due to dwindling sample size. This, however, would eliminate an element of bias along with ensuring that all operations were performed by the same surgeon.

The method of measurement of proprioceptive acuity in this study was using a modified Tornvall chair to measure RPP. Using the same apparatus, a significant difference in proprioceptive acuity has been previously identified between normal and osteoarthritic knees [15]. The results in our study ranged from 1°-18° in the injured knees to 1°-15° in the control knees (similar in range to Hassan et al.’s previous paper using the same apparatus), with standard deviations of 4.2 in the injured group and 3.8 in the control [15]. This may be explained either by individual’s wide variations in proprioceptive acuity or by inaccuracies in the measurement using the apparatus. A second published paper using the same apparatus subsequently failed to find a difference between radiographic osteoarthritis and controls [18].

There is debate on the most appropriate tool for measuring proprioception of the knee [19]. The two main methods of measurement are RPP (which measures joint position sense) and the threshold to detect passive motion (kinaesthesia). A number of tools have been developed to measure each of these. Grob et al. demonstrated no correlation between tests for kinaesthesia and joint position sense and no correlation between tests for joint position sense; however, a positive correlation was found between tests for kinaesthesia [19]. The method we chose in this study was based on the previous validation of the Tornvall chair [15].

The mean Lysholm score was 76±17, which is a lower score than in many of the other studies of multiligament knee injuries, but at a longer follow-up (mean: 83 months). Engebretsen et al.’s, Stannard et al.’s and Levy et al. studies published mean scores of 83, 89 and 85, respectively, at a mean follow-up of 60, 24 and 34 months [5,6,9]. The mean score of 76 is understandably less than the score recorded after isolated ACL reconstruction (which is usually between 85 and 90), as a multiligament knee injury is a much more serious injury [20]. This poorer outcome may in part be explained by the tendency with longer term retrospective studies to prejudice toward poorer outcomes. Patients with poorer results are more likely to attend an additional follow-up visit. With only 34 of the possible 81 patients being able to attend, the research clinic this could certainly be a confounder in this study.

## Conclusions

Our results suggest that proprioceptive acuity between the injured or control knee are equivalent irrespective of surgical techniques and that both reconstruction and repair can be validly used to achieve an acceptable result. This study has only touched the surface of outcomes for multiligament knee injuries and is a relatively small study, which we hope encourages future research.
